# Great saphenous vein leiomyosarcoma mimicking thrombosed aneurysm: A case report and review of the literature

**DOI:** 10.1016/j.jvscit.2023.101399

**Published:** 2023-12-16

**Authors:** Ammar Atieh, Hussein Allaw, Mohammad Ashouri, Mohammadreza Zafarghandi

**Affiliations:** a Department of Vascular and Endovascular Surgery, Sina Hospital, Tehran University of Medical Science, Tehran, Iran; b Department of Radiology, Namazi Hospital, Shiraz University of Medical Science, Shiraz, Iran

**Keywords:** Great saphenous vein, Leiomyosarcoma, Venous aneurysm

## Abstract

Vascular leiomyosarcoma LMS. is an extremely rare subgroup of LMSs. Fewer than 50 cases of LMS originating from the great saphenous vein have been reported. In 43% of reported cases, LMS was misdiagnosed clinically. In our case, the patient was initially misdiagnosed as having a thrombosed aneurysm. This misdiagnosis could be due to the rarity of great saphenous vein LMS cases, for which a high index of suspicion is needed, and because no specific radiologic findings have been established for diagnosing LMSs. Masses presenting along the course of vessels should be suspected for malignancy, which can be helpful in performing definitive surgery and avoiding multiple surgeries.

Primary malignant venous tumors are extremely uncommon, with leiomyosarcomas (LMSs) of the saphenous vein even rarer, accounting for ∼1 in every 1 million malignant tumors.^1^ Primary vascular LMSs (VLMSs) arise from the smooth muscle tissue of blood vessel walls. They are rarely diagnosed preoperatively.^2^ The tumor usually expands slowly and is difficult to diagnose before surgery because of its rarity and nonspecific symptoms. The most common clinical presentation reported in 60% of cases is a painless mass (Table). Additionally, superficial vein thrombophlebitis has been reported.^3^

**Table:** Reported cases of great saphenous vein (GSV) leiomyosarcoma

Investigator	Age, years	Sex	Mass size, mm	Location	Presenting symptom	Biopsy and treatment	Follow-up
Aufrecht,^15^ 1868	23	M	25	Below knee	Painful mass	NA	NA
Van Ree,^26^ 1919	42	F	NA	Below knee	Edema	NA	15 months; no LR or DM
De Weese et al,^27^ 1958	54	M	60	Above knee	Painful mass, edema	Incisional biopsy; excision of mass and femoral and external iliac veins	NA
Smout et al,^28^ 1960	76	F	90	Above knee	Painless mass	WLE; RT	NA
Dorfman et al,^29^ 1963	56	M	30	knee	Painless mass	NA	2 months; no LR or DM
Christiansen,^30^ 1964	68	F	45	Above knee	Painless mass	NA	6 months; no LR or DM
Allison,^31^ 1965	4	F	20	Above knee	Painless mass (thought to be enlarged LN)	Excision; re-excision, resection of anterior wall of femoral vein	6 months; no LR or DM
Hughes,^32^ 1973	53	F	25	Above knee	Painless mass	Excision	6 months; no LR or DM
Jernstrom et al,^33^ 1975	64	M	120	Above knee	Painless mass (thought to be of subcutaneous or muscular origin)	Intraoperative biopsy; excision	4 months; no LR or DM
Gross et al,^16^ 1975	46	M	50	Above knee	painless lump	Excision; near total thyroidectomy, RT (for neck metastases); excision of hypochondrium metastases	Thyroid metastasis after 3 years; after 8 months, hypochondrium metastasis
Stringer,^23^ 1977	39	M	NA	Above knee	Painful mass (thought to be neuroma)	Excision	NA
Stringer,^23^ 1977	36	F	NA	Above knee	Painful mass, edema	WLE; multiple surgeries for metastasis, CT, RT	Lung, scalp, bone, heart metastasis after 6 years; death after 11 years
Fischer et al,^34^ 1982	66	F	20	Above knee	Asymptomatic mass (thought to be metastatic)	Excisional biopsy; re-excision, lateral venorrhaphy	4 years; no LR or DM
Berlin et al,^11^ 1984	60	M	30	Above knee	Groin mass, femoral vein thrombosis	Incisional biopsy; extended hip joint disarticulation	Fatal PE after 1 month; autopsy showed lung and liver metastases
Leu et al,^35^ 1986	40	M	10	Above knee	Small nodule	Excision	18 years; no LR or DM
Humphrey et al,^12^ 1987	45	M	25	Above knee	Asymptomatic mass (thought to be venous thrombosis)	Excision; definitive surgery	4 years; no LR or DM
Welk et al,^36^ 1991	35	F	50	NA	NA	Excision, RT	11 months; no LR or DM
Song et al,^9^ 1991	54	F	50	Above knee	Painless mass (thought to be benign soft tumor)	Excision	NA
Dzsinich et al,^37^ 1992	70	F	NA	NA	NA	Resection	17 years; no LR or DM
Dzsinich et al,^37^ 1992	54	F	NA	NA	NA	Resection	Lung metastasis and death after 9 months
Saglik et al,^24^ 1992	61	F	60	Above knee	Painless mass (thought to be lipoma)	Excision; multiple surgeries for LR; CT for lung metastasis; WLE for LR; refused CT after second metastasis	LR after 6 months (several); lung metastases after 6 months; remission; LR after 18 months; lung metastases after 14 months; death after 8 months
Stallard et al,^2^ 1992	64	F	70	Above knee	Painless mass	Open biopsy; excision	NA
Byard et al,^38^ 1993	2	F	25	Above knee	Painless mass (thought to be fibromatosis)	Localized excision; WLE, LN dissection, and CT for LR; radical en bloc resection and CT for second LR	LR 1 year later; LR after 2 years; 9 years follow-up after last LR
Stambuk et al,^39^ 1993	48	M	41	Above knee	Painless mass	Excision; re-excision, RT	1 year; no LR or DM
Reix et al,^25^ 1998	64	M	50	NA	NA	Resection, CT	Metastases after 14 months (skin, lung, brain); no LR; died after 6 years
Marle et al,^1^ 2004	85	F	25	Above knee	Painful swelling (thought to be an enlarged LN)	WLE; refused adjuvant RT	2 months; no LR or DM
Le Minh et al,^6^ 2004	52	F	20	Above knee	Asymptomatic swelling (thought to be venous thrombosis)	Excisional biopsy; WLE, RT, CT	1 year; no LR or DM
Le Minh et al,^6^ 2004	66	M	50	Above knee	Asymptomatic swelling (thought that tumor was a subcutaneous lipoma)	Excision, RT	6 months; no LR or DM
El Khoury et al,^40^ 2006	60	M	30	Above knee	Painless mass (thought to be femoral lymphadenopathy, suspicious for lymphoma recurrence)	FNA; WLE	6 months; no LR or DM
Zhang et al,^21^ 2006	59	F	2 masses: 40 and 60	Above knee	Painful mass (“thrombus” of saphenous vein diagnosed by ultrasound)	Excision, RT	10 months; no LR or DM
Mammano et al,^41^ 2008	48	M	60	Above knee	Right inguinal mass	FNA; en bloc removal of mass, LN dissection, femoral vein reconstruction with PTFE	Lung metastasis 25 months after surgery; died 30 months after surgery
Yanagita et al,^42^ 2010	79	M	30	Above knee	Asymptomatic (PET for thymus cancer)	Excision; complete resection	NA
Bibbo et al,^8^ 2011	64	F	70	Below knee	Painless mass (thought to be ganglion cyst)	Intraoperative open biopsy (malignancy could not be ruled out); excision (positive margins); RT, WLE	12 months; no LR or DM
Werbrouck et al,^10^ 2013	57	M	26	Above knee	Painless mass	FNA; total resection	NA
Amato et al,^43^ 2013	72	M	60	Above knee	Painless mass (thought to be LN and thrombus)	Excision; radical excision, LN dissection, RT	Lung and bone metastasis after 6 months; died after 5 months
Fremed et al,^5^ 2014	59	M	34	Above knee	Lower extremity edema, extensive DVT	En bloc excision	6 months; no LR or DM
Lin et al,^14^ 2016	75	M	20	Below knee	Painful lump (thought to be mesenchymal tumor or thrombosed segment)	Localized excision; WLE	6 months; no LR or DM
Cangiano et al,^44^ 2016	65	F	20	Above knee	Painless mass (thought to be femoral lymphadenopathy)	FNA; en bloc WLE	10 months; no LR or DM
Macarenco et al,^19^ 2017	57	M	37	Below knee	Painless mass	Incisional biopsy; WLE, RT	3 years, 3 months; no LR or DM
Zhang et al,^45^ 2019	83	M	40	Below knee	Painful mass	Excision	6 months; no LR or DM
Naouli et al,^7^ 2019	45	M	30	Above knee	Painless swelling	WLE	6 months; no LR or DM
Güner et al,^46^ 2020	34	M	NA	NA	History of GSLMS; auscultation murmur	GSLMS (multiple surgeries); resection of cardiac metastasis	Cardiac metastasis after 3 years
Tresgallo-Parés et al,^18^ 2021	67	F	40	Above knee	Painless mass	Wide excision (positive margin); re-excision, RT	2 years; no LR or DM
Alkhaled et al,^4^ 2022	49	F	16	Above knee	Painful mass	Excision	6 months; no LR or DM
Dziekiewicz et al,^3^ 2022	61	F	2 masses: 100 and 50	Below knee	Superficial thrombophlebitis symptoms	Excision	1 year; no LR or DM

*CT,* Chemotherapy; *DM,* distant metastasis; *DVT,* deep vein thrombosis; *F,* female; *FNA,* fine needle aspiration; *GSLMS,* great saphenous vein leiomyosarcoma; *LN,* lymph node; *LR,* local recurrence; *M,* male; *NA,* not available; *PE,* pulmonary embolism; *PET,* positron emission tomography; *RT,* radiotherapy; *WLE,* wide local excision.

No specific laboratory or radiologic investigations are available that can help determine the definitive diagnosis. The only method available to confirm the diagnosis is histopathologic examination.^4^ In the lower limbs, the great saphenous vein (GSV) is the most common site of origin for primary VLMSs. In 80% of patients, a VLMS originates from the above the knee segment of the GSV (Table). The prognosis and treatment depend on the tumor size, tumor grade, and ability to perform wide local excision with a 2- to 3-cm margin.

## Case report

A 63-year-old woman presented with painless swelling in the groin. She was otherwise healthy and stated that the swelling had developed during the previous several months. On examination, her vital signs were normal, and a pulseless, painless swelling with normal color was noted at the medial aspect of her thigh. She had no previous medical issues or surgical interventions, and the laboratory study results were normal.

**Fig 1:**
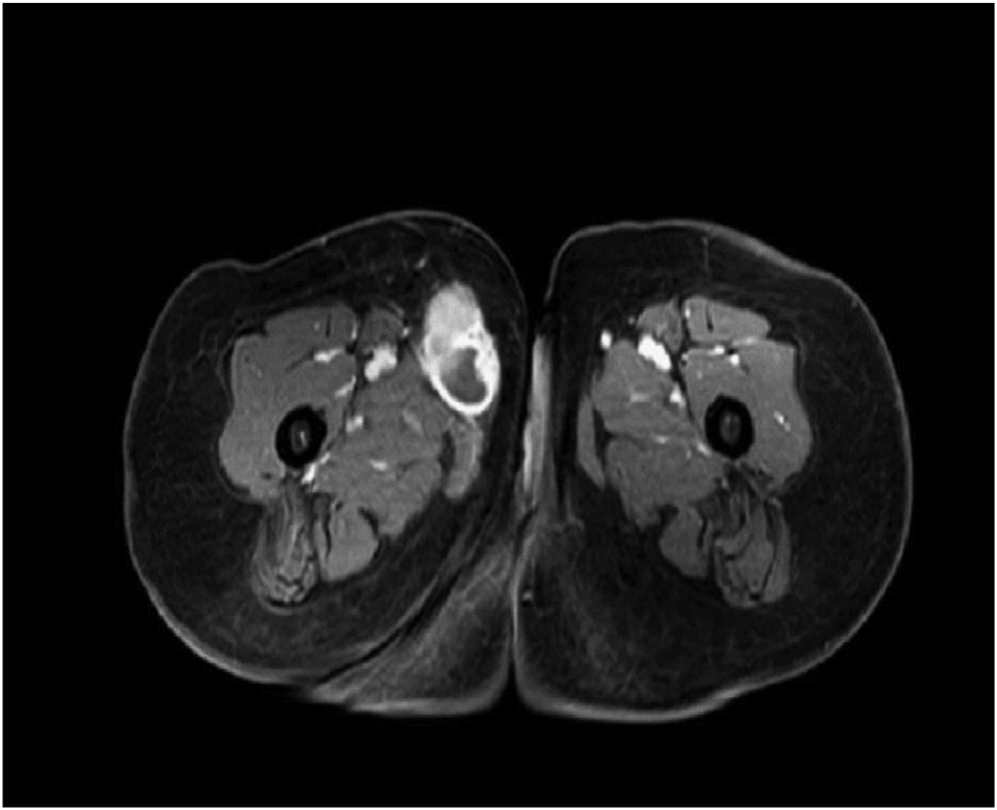
Axial view showing enhancing mass within great saphenous vein (GSV) with cystic spaces.

**Fig 2:**
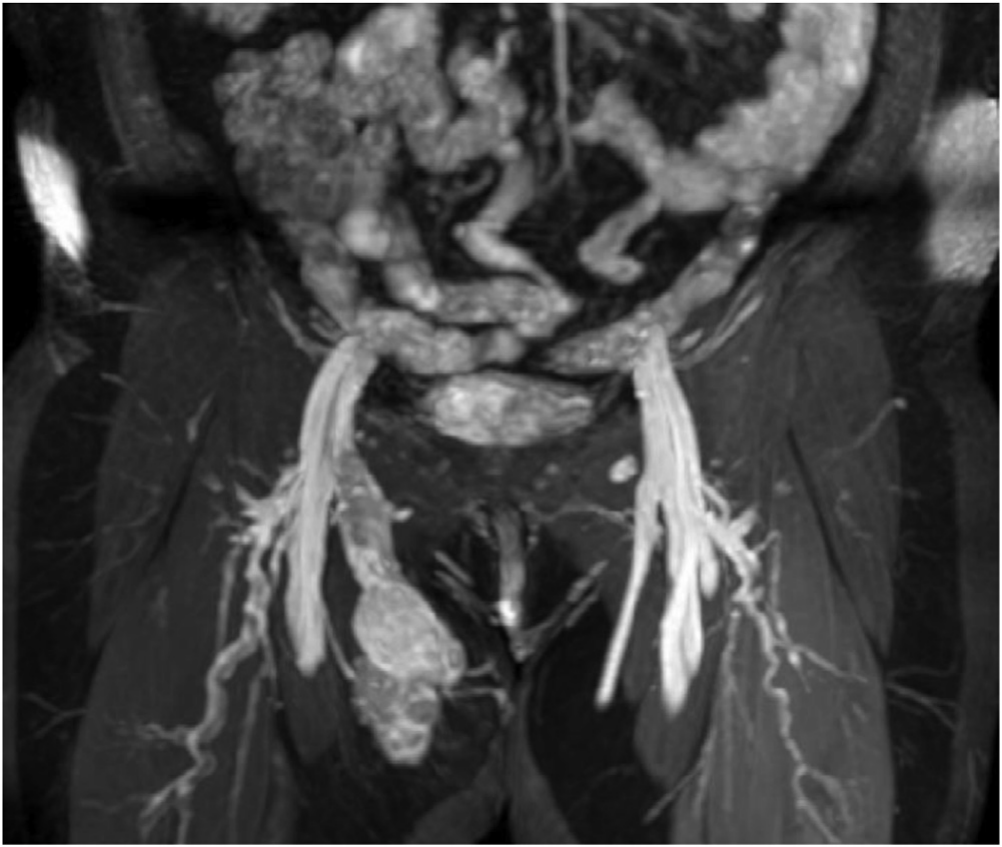
Coronal view showing extension of mass along course of great saphenous vein (GSV) into common femoral vein. Note mild enhancement of some portions of the mass, which led to the misdiagnosis. This was falsely reported as an aneurysmal dilation with internal thrombosis.

**Fig 3:**
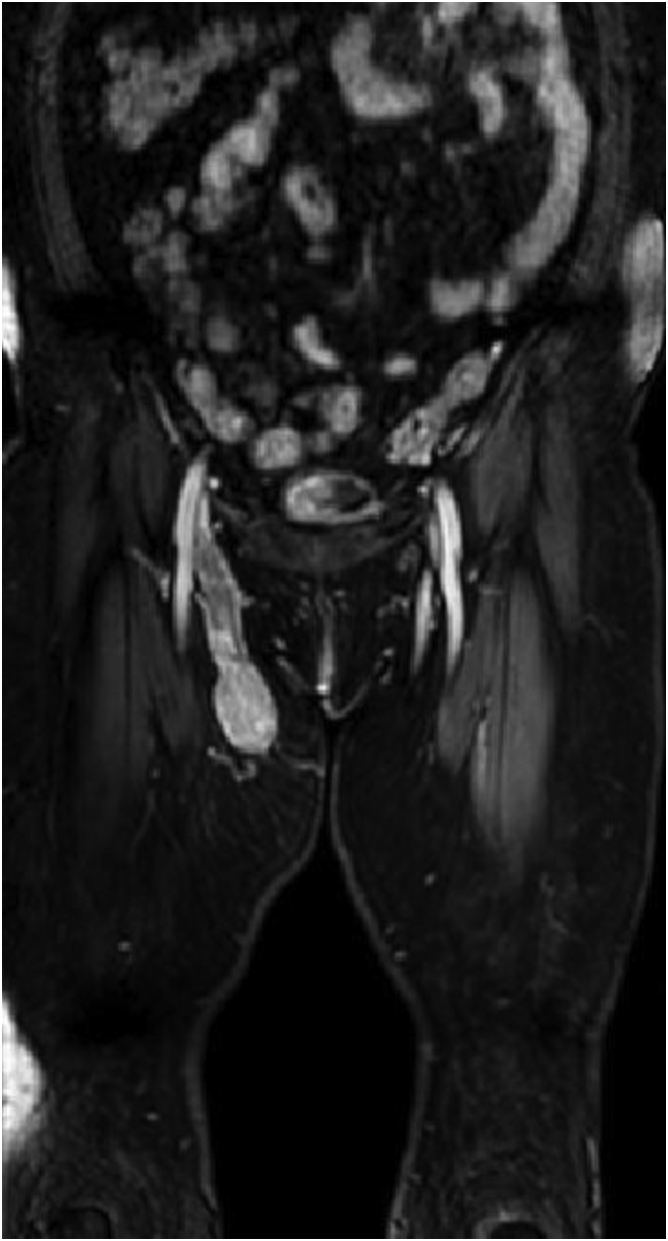
Coronal view of contrast-enhanced magnetic resonance imaging (MRI) showing an inhomogeneously enhancing mass within the proximal great saphenous vein (GSV) expanding the lumen. Less prominent enhancement is present in its superior portion, mimicking a filling defect and thrombosis.

Before being referred to our center, Doppler ultrasound revealed a thrombosed aneurysmal dilation of the GSV close to the saphenofemoral junction without blood flow. However, for a more detailed assessment of the alleged aneurysm and to screen for potential venous aneurysms occurring simultaneously, abdominal and extremity magnetic resonance venography (MRV) was performed. MVR demonstrated an intraluminal mass that was filling and expanding the GSV and slightly extending to the common femoral vein, with mild to moderate inhomogeneous enhancement and some cystic areas (Fig 1, Fig 2, Fig 3), in favor of thrombosed aneurysmal dilation of the GSV. The patient was scheduled for GSV aneurysm resection.

The patient received systemic heparinization before the procedure, and 5000 IU of heparin was administered during the procedure. During the operation, a firm mass measuring 6 cm was found within the saphenous vein, without extraluminal extension. The common femoral vein was first clamped distally to prevent potential embolism and then clamped proximally. The progression into the saphenofemoral junction was managed by applying digital pressure and flushing in an attempt to extract the intraluminal extension. The mass was resected. At 2 weeks postoperatively, histopathologic examination revealed an LMS originating from the GSV, with the distal margin (close to the saphenofemoral junction) involved by the tumor. The immunohistochemistry interpretation confirmed the previous pathology report. A metastasis workup was conducted before proceeding with another surgery. Chest computed tomography without contrast revealed bilateral lung metastases, evident as multiple bilateral small lung nodules scattered within the lung parenchyma. The patient was referred to an oncologist and is a candidate for wide local excision and adjuvant chemotherapy. The patient and institute provided written informed consent for the report of her case details and imaging studies.

## Discussion

### Presentation

VLMS is an extremely rare subgroup, accounting for <2% of LMSs.^5^, ^6^, ^7^, ^8^ LMSs are more commonly found in large veins and are rare in arteries,^9^ with localization in veins five times more common than in arteries.^6^, ^7^, ^8^ The inferior cava vein is the predominant venous location, accounting for almost one half of cases, followed by the pulmonary, renal, common femoral, saphenous, superior mesenteric, and ovarian veins and superior cava vein.^10^ Upper extremity vein sarcoma has also been reported.^11^ The first case of LMS of the inferior vena cava was reported in 1871 by Perl Tara.^12^^,^^13^ The GSV is the most frequent site of origin of LMSs in the lower extremities,^14^ with <50 cases reported since it was first described by Aufrecht^15^ in 1868. The size of VLMSs can vary greatly, with reported diameters ranging from 10 to 120 mm (mean, ∼43 mm). The age of patients with VLMS ranges from 2 to 85 years (median age, 57 years), with no gender predominance (Table).

### Diagnosis

In 43% of reported cases, LMS was not clinically suspected and was instead thought to be another condition, such as lipoma, neuroma, fibromatosis, venous aneurysm, enlarged lymph nodes, and others (Table). In some cases, radiologic and tissue studies were unable to provide a definitive diagnosis before surgery, resulting in multiple surgeries being performed (Table). The diagnosis of malignancy is not often made preoperatively.^16^ Based on the anatomic location, our clinical diagnosis initially suggested an enlarged femoral lymph node. Although magnetic resonance imaging (MRI) is highly accurate for diagnosing soft tissue tumors and is the preferred modality, for our patient, a GSV LMS was misdiagnosed as a thrombosed aneurysm using MRV and Doppler ultrasound. This might have been due to the lack of specific radiologic findings for LMS, the rarity of GSV LMS cases (requiring a high index of suspicion), the absence of usual MRI sequences (ie, T1, T2, gradient echo, diffusion-weighted imaging/apparent diffusion coefficient of water), and the mild to moderate enhancement of some portions of the mass on MRV (Figs 1and3). Aneurysm of the proximal GSV segment has been reported; however, it is uncommon. The signal intensity on MRI for venous thrombosis is generally increased on T1- and T2-weighted images, with clear delineation of the thrombus from the vascular wall, which shows a low signal intensity.^17^

The presence of an intraluminal mass in a vein on MRI should suggest the diagnosis of venous LMS, which requires definitive operative excision. The preoperative diagnosis of a noncaval venous LMS allows for planning of resection without biopsy, reducing the risk of seeding by hemorrhage that can occur with incisional biopsy.^2^ There is a belief that incisional biopsy could increase the risk of recurrence and metastasis by spreading tumor cells. Therefore, the significant advantage of avoiding incisional biopsy lies in terms of reducing seeding and minimizing the risk of recurrence and metastasis. Avoiding an incisional biopsy can be a significant advantage for the patient.^11^

More than 25% of reported cases underwent tissue diagnosis before surgery (Table). When diagnostic confirmation is necessary, direct tissue evaluation should be performed.^18^ This can be done through needle or core biopsies, followed by an open biopsy, if needed. A histologic diagnosis is crucial in determining the extent of resection, which can help in performing single-stage surgery with wide local excision instead of multiple surgeries. If the needle biopsy results are inconclusive, an incisional biopsy specimen will be necessary to guide the radiologic evaluation and confirm the presence of a superficial VLMS before surgical treatment.^19^ An excisional biopsy can be performed for primary lesions <3 cm, with incisional biopsy used for larger masses >3 cm.^12^^,^^20^

### Treatment

Currently, the mainstay treatment of venous LMSs appears to be wide excision of the tumor, combined with adjuvant radiotherapy.^21^ The goal of any surgical management plan is local disease control with preservation of limb function. The operative treatment of venous LMS involves wide surgical excision with a resection margin of 2 to 3 cm.^12^^,^^22^ Radical surgical excision locally appears to be appropriate for both primary lesions and isolated metastases.^23^

Local recurrence is rare, and distant metastases to the thyroid, heart, scalp, bone, liver, skin, brain, and lungs have been frequently reported, with the lungs the most common site (Table). If any form of adjuvant therapy is used, it is usually radiotherapy, with chemotherapy reserved for cases in which distant metastasis occurs.^22^^,^^24^ Evidence supports the use of radiotherapy for the local management of soft tissue sarcomas in general, especially for larger lesions,^12^^,^^25^ and for high-grade tumors.^14^ For smaller soft tissue sarcomas measuring <5 cm, adjunctive radiotherapy might not be necessary due to their significantly lower recurrence rate compared with that of larger lesions^8^

## Conclusions

This review should guide the approach to masses presenting along the course of vessels. Any intraluminal or extraluminal mass that presents close to a vessel should be suspected for malignancy. Due to their rarity, nonspecific symptoms, and nonspecific radiologic findings, VLMSs can be misdiagnosed. Therefore, a high level of suspicion is necessary. Also, it would be more helpful the for diagnosis, if MRI sequences (ie, T1, T2, gradient echo, diffusion-weighted imaging/apparent diffusion coefficient of water). were obtained, in addition to MRV. A diagnosis before the procedure could aid in performing definitive surgery and avoiding multiple surgeries.

## Disclosures

None.
